# Hepatitis B knowledge, perceptions and practices in the French general population: the room for improvement

**DOI:** 10.1186/1471-2458-13-576

**Published:** 2013-06-13

**Authors:** Cécile Brouard, Arnaud Gautier, Leïla Saboni, Christine Jestin, Caroline Semaille, Nathalie Beltzer

**Affiliations:** 1 Department for infectious diseases, French Institute for Public Health Surveillance (InVS), 12, rue du Val d’Osne, Saint-Maurice Cedex, 94415, France; 2 National Institute for Prevention and Health Education (Inpes), 42, Boulevard de la Libération, Saint-Denis Cedex, 93203, France; 3 Paris Region Health Observatory, 43, rue Beaubourg, Paris, 75003, France

## Abstract

**Background:**

Little is known about the knowledge, perceptions and prevention practices of the French general population with respect to Hepatitis B virus (HBV) infection. This article describes this population’s knowledge of HBV, their perceptions of the disease, and associated screening and vaccination practices. It compares these indicators with those observed in the same population for HIV, an infection with a chronic course and transmission modes resembling those of HBV.

**Methods:**

A module on hepatitis B was added into the HIV KABP (Knowledge, Attitudes, Beliefs and Practices) survey which was carried out telephonically in 2010 among a random sample of 9,014 individuals aged between 18–69 and living in metropolitan France.

**Results:**

Compared with HIV, the general population was less aware that needle exchange during intravenous drug use and sexual relationships are HBV transmission modes (HBV: 89.9% and 69.7%; HIV: 99.1% and 99.4%). The fear of both illnesses was similar at 20.3%. The individual perceived risk of infection was higher for HBV than for HIV with, respectively, 60.8% and 40.3% of respondents believing they had an equal or greater risk of being infected than the average person. However, the percentage of those reporting HBV screening during their lifetime (27.4%) was half that for HIV screening (61.4%). In multivariate analysis, HBV screening was reported more often by individuals born in areas with high HBV endemicity (OR = 2.1 [95% CI: 1.5-2.9]) than by those born in low HBV endemicity areas, and more often by those who reported they had taken drugs intravenously during their lifetime (OR = 2.2 [95% CI: 1.2-4.2]) than those who did not report such behavior. Almost one in two respondents (47%) reported HBV vaccination. The intermediate or high endemicity groups did not report vaccination more often than those born in low endemicity areas nor did those reporting intravenously drug use compared with those who did not.

**Conclusions:**

This study highlights very contrasting levels of knowledge, perceptions and practices regarding HBV and HIV in the French general population. Our results demonstrate the need to improve the general and high-risk populations’ knowledge of HBV, in particular concerning sexual transmission, in order to improve screening and vaccination practices.

## Background

Hepatitis B is a vaccine-preventable infection. It can be transmitted sexually, by blood, from mother to child during childbirth and by family contact [1]. For the most part asymptomatic, patients can nevertheless develop serious liver conditions (cirrhosis, hepatocellular carcinoma) which sometimes lead to death. Worldwide, chronic hepatitis B virus (HBV) infection concerns 370 million people, that is 5% to 7% of the world’s population, and is responsible for more than 500,000 deaths per year [1].

France is a country with low HBV endemicity, the prevalence for chronic infection in 2004 being estimated at 0.65% in the general population aged between 18–80 years old [2]. An estimated 1,300 deaths were attributable to the disease annually [3]. In France, the following principal factors are associated with HBV infection: being born in a country where there is an intermediate or high HBV endemicity [4], intravenous drug use, defining oneself as homosexual and being in an economically and socially vulnerable situation [2]. Migrants represent approximately 8% of the general French metropolitan population (i.e. mainland France and the island of Corsica). A quarter of these migrants come from countries where HBV endemicity is high (14% Asia, 12% Sub Saharan Africa) [5]. Accordingly, HBV screening and vaccination are recommended for those at highest risk, notably sexual partners, close family and friends of HBV-infected individuals (whether acute or chronic), people who have multi-partner sexual relationships, migrants originating from areas with a high level of endemicity, parenteral drug users, health professionals, and others [6-8]. Moreover screening has been obligatory for pregnant women since 1992 [9], and vaccination has been recommended for infants since 1995 with a catch-up vaccination recommended for children and adolescents [6].

Despite the continued increase in hepatitis B screening since the 2000s [10], its efficacy remains insufficient. In 2004, more than one in two chronically infected persons were unaware of their status [2]. Moreover, anti-HBV vaccination coverage has remained low, especially since 1998, due to the suspicion of a link between anti-HBV vaccination and demyelinating diseases [11]. In 2003–4 and 2010, coverage was estimated at 42% for 15-year-old adolescents and 65% for 24-month-old children, respectively [12].

Although the principal epidemiological indicators are well documented, French [13-15] and international data [16,17] on the general population’s knowledge, perceptions and practices regarding hepatitis B are very fragmented. Yet these same indicators are essential for a better understanding of the mechanisms which can hinder or drive prevention behaviors and improve public health policy. Accordingly, the addition of a section on hepatitis B to the KABP 2010 survey, which focused primarily on the knowledge, attitudes, believes and practices of the French general population regarding HIV [18], provided us with the opportunity to measure, for the first time, these indicators for hepatitis B.

The objective of this paper is to present the French general population’s knowledge of the transmission modes of HBV, their perceptions regarding the disease and their practices with respect to screening and vaccination, by comparing these indicators with those for HIV, as the transmission modes and chronic evolution of the two diseases closely resemble each other.

## Methods

The KABP 2010 survey, approved by the National Data Protection Authority, was carried out anonymously over the telephone after oral consent for participation in the study [18]. Selection of the individual to be interviewed was carried out by random sampling in two phases: after a random generation of two samples of telephone numbers - one landline, the other mobile - one individual was randomly drawn from all the eligible people in that household (landline) or from all the eligible principal users of that phone number, if there was more than one user (mobile). All persons who could speak French, were aged between 18 and 69 and resident in metropolitan France were eligible. In order to take into account the probability of being selected and to keep the structure similar to that of the national population, the sample was first weighted for numbers of eligible adults in households and for the distribution of listed and unlisted landline and mobile phones. It was then adjusted for sociodemographic data gathered in the 2006 national census.

The data collected for hepatitis B, just as those collected for HIV/AIDS, focused on the interviewed person’s knowledge of transmission modes, perceptions and fears regarding these infections, knowledge of someone in his/her circle of family or friends being infected by HBV or HIV, and whether he/she had been screened for HBV or HIV or vaccinated against HBV. These indicators reflecting knowledge, perceptions and practices regarding HBV were compared with those for HIV. Furthermore the following indicators were examined in multivariate analysis using logistic regression: “having a good level of knowledge on the transmission modes of HBV”, “considering oneself to be at a higher risk of HBV contamination with respect to the average person”, “self-reported screening for HBV during one’s lifetime”, and “self-reported vaccination against HBV during one’s lifetime”. A good level of knowledge about the transmission modes of HBV was defined as providing a correct response to each of the following four questions: answering “yes” for the three questions worded as “Can transmission occur”…“during sexual relations without a condom?”, “when sharing a needle during drug injection?”, and “during pregnancy?” and answering “no” to the item “Can transmission occur from a mosquito bite?”.

Individuals were grouped according to the level of endemicity of HBV in their country of birth [4]. Having taken drugs intravenously was defined as reporting to have injected drugs at least once during one’s lifetime. Analyses were carried out using the Stata® 11.0 statistical software package (Stata Corporation, College Station, TX).

## Results

Final rates of participation for the survey were 67.2% and 65%, for landline and mobile phones respectively. In total, 9,014 people were interviewed: 5,012 on a landline and 4,002 on a mobile phone. The principal characteristics of the sample are outlined in Table 1.

**Table 1:** Socio-demographic characteristics of the sample, KABP survey, France, 2010

	**n **^**1**^	**% **^**2**^
Gender		
Men	4,074	48.9
Women	4,940	51.1
Age (in years)		
18–30	2,130	24.6
31–44	2,909	33.2
45–54	1,916	22.8
55-69	2,058	19.4
Educational Level		
No educational qualification	989	11.2
CAP/BEP/BEPC (vocational diplomas)	2,744	31.9
Secondary school certificate	1,687	19.1
Higher education qualification	3,573	37.8
Net monthly income per household		
<2,000 €	2,821	31.0
2,000–2,999 €	2,696	29.9
≥ 3,000 €	3 311	37.0
Did not wish to share this information/ Did not know	186	2.1
Area of residence		
Outside the Ile-de-France administrative district	6,598	80.6
Ile-de-France administrative district (which includes Paris)	2,416	19.4
Living in a couple or a stable relationship		
No	2,117	21.9
Yes	6,883	78.1
HBV endemicity in country of birth^3^		
Low	8,120	90.6
Intermediate	667	7.1
High	224	2.3
Intravenous drug use in one’s lifetime		
No	8,962	99.4
Yes	52	0.6
**Total**	**9,014**	**100**

^*1*^*raw data. Some data were missing for age (n = 1), educational level (n = 21), living in couple or a stable relationship (n = 14) and country of birth (n = 3).*

^*2*^*weighted and adjusted data.*

^*3*^*Countries with a low level of endemicity (prevalence of HBsAg <2%): Metropolitan France, Northern and Western European countries, North America, Pacific Islands; countries with an intermediate level of endemicity (prevalence of HBsAg between 2 and 8%): French Overseas Administrative Districts and territories, Eastern and Southern Europe, North Africa, the Middle-East, the Indian subcontinent, South America; countries with a high level of endemicity (prevalence of HBsAg > 8%): Sub-Saharan Africa, Asia.*

### Knowledge

Among the people interviewed, 96.1% declared having already heard of hepatitis B. Amongst these, 89.9% knew that HBV can be transmitted by needle-sharing while taking drugs, 79.1% that transmission can occur during pregnancy and 69.7% that transmission can occur during unprotected sexual intercourse (Table 2). Women were more aware of the possibility of transmission during pregnancy than men (81.3% *vs.* 76.6%, p < 10^-3^). The 18–30 year-old (74.6%) and 31–44 year-old (73.4%) populations were more aware of sexual transmission than was the 45–69 year-old population (63.9%, p < 10^-3^).

**Table 2:** Main reported indicators of knowledge, perceptions and behaviors with regard to HBV and HIV/AIDS, KABP survey, France, 2010

	**Hepatitis B**	**HIV/AIDS**
	**(n = 9,014)**	**(n = 9,014)**
	**% (95% CI)**	**% (95% CI)**
**Knowledge of transmission modes**^1^		
Believing that Hepatitis B/AIDS transmission is possible:		
during unprotected sexual intercourse (1)	69.7 (68.6-70.8)	99.4 (99.2-99.6)
through the sharing of needles while injecting drugs^2^ (2)	89.9 (89.2-90.6)	99.1 (98.9-99.3)
from mosquito bite (3)	26.0 (25.0-27.1)	24.5 (23.5-25.5)
during pregnancy (4)	79.1 (78.1-80)	-^4^
% of correct answers to questions (1), (2) and (3) ^**3**^	46.5 (45.3-47.6)	72.3 (71.3-73.3)
% of correct answers to questions (1), (2), (3) and (4) ^**3**^	38.4 (37.2-39.5)	-^4^
Knowing someone in circle of family and friends who was infected ^**5**^		
Yes	21.4 (20.5-22.3)	12.6 (11.9-13.4)
Perhaps	-	8.8 (8.2-9.4)
No	77.3 (76.3-78.2)	78.4 (77.5-79.3)
Did not know	1.3 (1.1-1.6)	0.16 (0.08-0.3)
**Perceptions**		
Fear of the disease (Viral hepatitis / AIDS) for oneself		
Very	9.8 (9.1-10.5)	13.4 (12.6-14.2)
Quite	10.5 (9.8-11.2)	6.9 (6.3-7.4)
Not very	39.4 (38.3-40.5)	24.6 (23.6-25.6)
Not at all	39.5 (38.4-40.6)	55.1 (54.0-56.3)
Did not know	0.8 (0.6-1.0)	0.04 (0.01-0.11)
Perception of the risk of contracting Hepatitis B /HIV with respect to the average person		
Greater risk	7.6 (7.0-8.2)	3.8 (3.4-4.2)
Similar risk	53.2 (52.1-54.4)	36.5 (35.4-37.6)
Less risk	29.9 (28.9-31.0)	37.5 (36.4-38.7)
No risk	7.4 (6.8-8.0)	21.9 (21.0-22.9)
Did not know	1.9 (1.6-2.4)	0.3 (0.2-0.4)
**Practices**		
Hepatitis B /HIV screening during one’s lifetime		
Yes, once	17.4 (16.6-18.3)	31.5 (30.4-32.5)
Yes, more than once	10.0 (9.4-10.7)	29.9 (28.8-31.0)
No	67.8 (66.7-68.8)	38.1 (37.0-39.2)
Did not know	4.8 (4.3-5.2)	0.5 (0.4-0.7)
Hepatitis B vaccination		
Yes	47.0 (45.9-48.2)	-
No	43.9 (42.8-45.1)	-
Did not know	9.0 (8.4-9.7)	-

95% CI: 95% confidence interval.

^*1*^*Only those people who reported that they had already heard of hepatitis B (n = 8,686) were asked questions about knowledge of hepatitis B transmission modes; all participants in the study group were asked all other questions (n = 9,014).*

^*2*^*The wording of questions was different depending on the virus, as follows: “Sharing a needle while injecting drugs” for HBV and “When injecting using a needle that has already been used” for HIV.*

^*3*^*The correct answer was “yes” for questions (1), (2) and (4) and “no” for question (3).*

^4^*These questions were not asked for HIV.*

^*5*^*The wording of questions was different depending on the virus, as follows: “Do you personally know one or more people in your circle of family and friends who currently has or who had Hepatitis B?” / “Do you personally know one or more people in your circle of family and friends who is seropositive or who has AIDS?. The choice of answers was also different (yes, no, did not know for HBV; yes, perhaps, no, did not know for HIV).*

Transmission by sexual relations and intravenous drug-use was less known for HBV (69.7% and 89.9%) than for HIV (99.4% and 99.1%). The proportion of those declaring “not to know” about these two transmission modes was higher for HBV (approximately 4%) than for HIV (approximately 0.2%). The proportion of people incorrectly stating that transmission was possible from a mosquito bite was similar for both diseases (HBV: 26%; HIV: 24.5%). In total, 46.5% of those interviewed correctly responded to these three questions (i.e. sexual relations, intravenous drug use and mosquito-bite transmission) regarding HBV versus 72.3% for HIV. This proportion fell to 38.4% when the survey question regarding “transmission of HBV during pregnancy” was taken into account.

In multivariate analysis, a good level of knowledge of HBV transmission modes was found more often in women than in men, in the 18–30 year-old population than in the 45–54 and 55–69 year-old populations, in people who had educational qualifications (compared with those without qualifications), in those whose net monthly household income was greater than 3,000 Euros (compared with less than 2,000 Euros), in those who did not live in a couple or in a stable relationship, and in those who reported to know someone infected with HBV in their circle of family and friends (Table 3).

**Table 3:** **Factors associated with a good level of knowledge about HBV transmission modes**^**1**^**, KABP survey, France, 2010 (multivariate analysis)**

	**%**	**Adjusted OR**	**CI 95%**
Gender			
Men	35.1	1	
Women	41.4	**1.3**	**1.2-1.5**
Age (in years)			
18–30	42.6	1	
31–44	42.5	1.0	0.9-1.1
45–54	34.9	**0.8**	**0.6-0.9**
55-69	30.0	**0.6**	**0.5-0.7**
Educational level			
No educational qualification	26.1	1	
CAP/BEP/BEPC (vocational diplomas)	32.8	**1.3**	**1.1-1.6**
Secondary school certificate	38.6	**1.5**	**1.2-1.8**
Higher education qualification	46.2	**2.0**	**1.6-2.4**
Net monthly income per household			
<2,000 €	35.5	1	
2,000–2,999 €	37.1	1.0	0.9-1.2
≥ 3,000 €	42.3	**1.2**	**1.1-1.3**
Did not wish to share this information/ Did not know	25.4	**0.7**	**0.4-0.96**
Area of residence			
Outside the Ile-de-France administrative district	37.4	1	
Ile-de-France administrative district (which includes Paris)	42.2	1.1	0.99-1.2
Living in a couple or a stable relationship			
No	39.9	1	
Yes	38.0	**0.9**	**0.8-0.98**
Knowing someone in circle of family and friends who was infected			
No	37.4	1	
Yes	41.9	**1.2**	**1.1-1.3**
HBV endemicity in country of birth^2^			
Low	38.7	1	
Intermediate or high	35.7	0.9	0.8-1.1
Intravenous drug use in one’s lifetime			
No	38.4	1	
Yes	29.0	0.8	0.4-1.6

95% CI: 95% confidence interval.

^*1*^*A good level of knowledge was defined as providing the correct response to the 4 questions asked about HBV transmission modes, as follows: “yes” for the items “sexual relations without a condom”, “through the sharing of needles while injecting drugs”, and “during pregnancy”; “no” for the item “from a mosquito bite”.*

^*2*^*See the footnote 3 in Table*1*.*

It should be noted that the percentage of respondents reporting to know someone in their circle of family and friends who was infected with HBV (21%) was higher than that found for HIV (12.4%) (Table 2). This proportion was greater for those respondents born in zones of intermediate or high HBV endemicity than for those born in zones of low endemicity (25.0% *vs.* 21.0%, p < 0.05). It was also greater for those who declared intravenous drug use during their lifetime than for those who did not (36.1% *vs.* 21.3%, p < 0.05).

### Perceptions

Among the various risks and diseases which participants reported that they were “quite” or “very” afraid of, viral hepatitis and AIDS yielded similar results (20.3%). These values were in fifth position after cancer (59.4%), road accidents (58.7%), senile dementia (39.8%) and heart disease (38.8%), and before sexually transmissible diseases (excluding HIV) (17.3%) and tuberculosis (11.6%). Nevertheless, the proportion of those not having any fear about HIV (55.1%) was higher than that for viral hepatitis (39.5%) (Table 2).

With, respectively, 7.6% and 53.2% of respondents considering they had “a greater risk than” and “the same risk as” the average person of being contaminated by HBV (*vs.* 3.8% and 36.5% respectively for HIV), the perception of the risk of contamination was higher for HBV than for HIV (Table 2). In multivariate analysis, the factors associated with considering oneself more at risk of HBV contamination than the average person were: being aged between 31–44 or 45–54 (in contrast to 18–30), having no educational qualification, not living in a couple or in a stable relationship and knowing someone infected with HBV (Table 4). Perception of being at a greater risk than the average person was not associated with the level of knowledge of HBV transmission modes.

**Table 4:** Factors associated with the perception of being at greater risk of HBV infection with respect to the average person, KABP survey, France, 2010 (multivariate analysis)

	**%**	**Adjusted OR**	**CI 95%**
Gender			
Men	7.7	1	
Women	7.4	0.9	0.8-1.1
Age (in years)			
18–30	5.1	1	
31–44	8.4	**1.7**	**1.3-2.3**
45–54	10.8	**2.2**	**1.7-2.9**
55-69	5.5	1.0	0.7-1.3
Educational level			
No educational qualification	9.8	1	
CAP/BEP/BEPC (vocational diplomas)	7.9	**0.7**	**0.5-0.95**
Secondary school certificate	5.8	**0.6**	**0.4-0.8**
Higher education qualification	7.5	**0.7**	**0.5-0.9**
Net monthly income per household			
<2,000 €	7.4	1	
2,000–2,999 €	7.5	1.1	0.9-1.4
≥ 3,000 €	7.5	1.1	0.8-1.4
Did not wish to share this information/ Did not know	11.5	**1.8**	**1.03-3.2**
Area of residence			
Outside the Ile-de-France administrative district	7.6	1	
Ile-de-France administrative district (which includes Paris)	7.4	0.9	0.8-1.2
Living in a couple or a stable relationship			
No	8.3	1	
Yes	7.3	**0.8**	**0.6-0.95**
Knowing someone in circle of family and friends who was infected			
No	6.9	1	
Yes	9.9	**1.5**	**1.2-1.8**
Having a good level of knowledge about HBV transmission modes			
No	7.4	1	
Yes	7.9	1.1	0.9-1.3
HBV endemicity in country of birth^1^			
Low	7.5	1	
Intermediate or high	8.3	1.1	0.8-1.4
Intravenous drug use in one’s lifetime			
No	7.6	1	
Yes	7.9	0.7	0.2-2.4

95% CI: 95% confidence interval.

^*1*^*See the footnote 3 in Table*1*.*

### Screening and vaccination practices

#### Hepatitis B screening

Just over a quarter of respondents declared that they had been screened for hepatitis B during their lifetime (17.4% on one occasion, 10.0% on more than one occasion) compared with 61.4% for AIDS (31.5% on one occasion, 29.9% on more than one occasion) (Table 2). Although women did not report screening for HBV more often than men (27.8% and 27.1% respectively), those women with at least one child born after 1992 (the year when obligatory HBV screening during pregnancy was implemented in France) reported screening more often than did those with no child born after 1992 (32.9% *vs.* 24.3%, p < 10^-4^). The younger the last child, the higher was this proportion, reaching 39.9% in women who had a child less than one year old. Reporting previous hepatitis B screening increased with increasing HBV endemicity in the country of birth, from 26.8% to 30.1% and 45.1% for those born, respectively, in low, intermediate and high endemicity zones.

Multivariate analysis highlighted that reporting to have been screened for hepatitis B was more frequent in the following situations: as the educational level achieved increased, in the 31–44 year-old population (compared with the 18–30 year-old one), in those who considered themselves at greater risk than the average person, in those who knew an infected person, in those born in areas with high HBV endemicity (when compared with those born in low endemicity areas), in those who declared intravenous drug use in their lifetime and in inhabitants of Île-de-France administrative district (Table 5). It was also associated with a good level of knowledge of transmission modes and with the fact of reporting to be vaccinated against HBV.

**Table 5:** Factors associated with HBV screening during one’s lifetime and anti-HBV vaccination, KABP survey, France, 2010 (multivariate analysis)

	**HBV screening**			**Anti-HBV vaccination**		
	**%**	**Adjusted OR**	**CI 95%**	**%**	**Adjusted OR**	**CI 95%**
Gender						
Men	**27.1**	**1**		45.1	1	
Women	**27.8**	**0.95**	0.9-1.1	48.9	**1.2**	**1.1-1.3**
Age (in years)						
18–30	26.0	1		62.3	1	
31–44	32.6	**1.3**	**1.2-1.5**	54.5	**0.7**	**0.6-0.7**
45–54	26.5	1.2	0.97-1.4	36.4	**0.3**	**0.3-0.4**
55-69	21.7	1.1	0.9-1.3	27.4	**0.2**	**0.2-0.3**
Educational level						
No educational qualification	18.2	1		33.7	1	
CAP/BEP/BEPC (vocational diplomas)	23.4	**1.4**	**1.04-1.6**	41.3	1.1	0.9-1.3
Secondary school certificate	26.1	**1.5**	**1.1-1.8**	49.5	**1.2**	**1.01-1.5**
Higher education qualification	34.2	**1.8**	**1.5-2.3**	54.4	**1.5**	**1.2-1.8**
Net monthly income per household						
<2,000 €	25.6	1		45.8	1	
2,000–2,999 €	26.6	0.9	0.8-1.1	47.4	1.0	0.9-1.2
≥ 3,000 €	30.4	0.9	0.8-1.1	48.2	1.0	0.9-1.2
Did not wish to share this information/ Did not know	16.2	**0.6**	**0.4-0.9**	40.5	0.9	0.6-1.3
Area of residence						
Outside the Ile-de-France administrative district	26.2	1		46.1	1	
Ile-de-France administrative district (which includes Paris)	32.5	**1.2**	**1.04-1.3**	50.9	1.0	0.9-1.2
Living in a couple or a stable relationship						
No	25.4	1		48.0	1	
Yes	28.1	1.1	0.98-1.3	46.8	1.0	0.9-1.2
Knowing someone in circle of family and friends who was infected						
No	23.7	1		46.5	1	
Yes	41.1	**2.1**	**1.9-2.4**	49.0	1.0	0.9-1.2
Having a good level of knowledge about HBV transmission modes						
No	23.9	1		44.3	1	
Yes	33.6	**1.4**	**1.3-1.6**	51.7	1.1	0.97-1.2
Perception of being at greater risk of HBV infection with respect to the average person						
No	25.6	1		45.5	1	
Yes	50.1	**2.5**	**2.1-3.1**	66.1	**2.2**	**1.8-2.7**
HBV endemicity in country of birth^1^						
Low	26.8	1		46.8	1	
Intermediate	30.1	1.2	0.9-1.4	47.3	1.1	0.9-1.4
High	45.1	**2.1**	**1.5-2.9**	56.1	1.2	0.8-1.6
Intravenous drug use in one’s lifetime						
No	27.4	1		47.0	1	
Yes	45.6	**2.2**	**1.2-4.2**	52.1	1.2	0.6-2.4
HBV screening during one’s lifetime						
No	-	**-**	**-**	40.3	1	
Yes				64.8	**2.4**	**2.2-2.7**
Anti-HBV vaccination						
No	18.3	1		**-**	-	**-**
Yes	37.8	**2.4**	**2.2-2.7**			

95% CI: 95% confidence interval.

^*1*^*See the footnote in Table*1*.*

Amongst those who reported that they had already been screened, 3.7% [95% CI: 2.9-4.5%] reported that they currently had or previously had hepatitis B (men: 4.2%: women: 3.2%; p > 0.05). This proportion increased with age (1.8%, 2.4%, 4.2% and 9.1%, respectively, for the 18–30, 31–44, 45–54 and 55–69 year-old populations, p < 10^-4^) and with the level of HBV endemicity in the country of birth (3.4%, 4.8%, 9.5% respectively for low, intermediate and high endemicity levels, p < 10^-2^). It was also higher in people who declared intravenous drug injection in their lifetime than in those who did not (28.6% vs. 3.5%, p < 10^-4^).

#### Vaccination against hepatitis B

Almost half of the respondents (47%) declared that they had been vaccinated against hepatitis B (irrespective of the number of vaccination doses administered) while 9.0% declared that they did not know if they had been vaccinated or not (Table 2). Independently of the other factors, anti-HBV vaccination was more frequently reported by women, by the 18–30 year-old population (with respect to higher age groups), by people with an educational level equal to or higher than a secondary school certificate (with respect to those with no educational qualification), by those who believed themselves to be at greater risk of contamination than the average person and by those who declared that they had been screened during their lifetime (Table 5). It was not significantly associated with the level of HBV endemicity in the country of birth or with intravenous drug injection during one’s lifetime.

## Discussion

This study enabled us to document, for the first time in France, the knowledge, perceptions and practices of the general population with regard to hepatitis B and to compare these same factors with those for HIV. Although, as our results show, the transmission modes for the former were certainly less known than those for the latter – especially regarding HBV infection through sexual transmission, unknown to 1 in 3 respondents - the fear of viral hepatitis seemed to be as strong as that of HIV and the perception of the risk of contamination with respect to the average person seemed to be higher for the former than the latter. It is true that today, in France, the epidemiological context is less favorable to HBV than to HIV with, respectively, an estimated prevalence of 280,000 infected people [2] versus 152,000 [19]. However, in the present study, the proportion of those reporting to have been screened for HBV during their lifetime was less than half that for HIV (27% *vs.* 61%).

The contrasting results found in the present study for these two infections whose characteristics (transmission modes, chronic course) are closely related, can be partly explained by the exceptional communication of the issues surrounding HIV, in particular the large scale worldwide media prevention campaigns right from the moment of its discovery. Despite two successive national prevention programs (2002–2005, 2009–2012 [20]) and several information campaigns (documents, website, movies…) targeted to migrants and men who have sex with men, communication campaigns on HBV and resources allocated to HBV remained modest in comparison with HIV. The fact that a vaccination exists for HBV but not for HIV may also play a part in some of the differences observed, notably the lower levels of reported HBV screening. Nevertheless, multivariate analyses on the associations between knowledge, perceptions and practices regarding HBV highlighted results which were very close to those found for HIV [18], with the exception, for HIV, of the association between an increased perception of being at greater risk of contamination than the average person and a low level of knowledge, something which was not found for HBV.

Although HBV can be transmitted sexually (in the same way as HIV), a large proportion of the general population were still unaware of this. Only 70% of the respondents reported that they knew this fact, which is similar to the percentages found in other European studies: 63% and 78%, respectively, in Germany and the Netherlands [21]. In contrast, transmission by sharing needles when injecting drugs was better known (90% in our study). Indeed, this difference in knowledge about these two transmission types has already been observed in other countries. For example, in one German study, these values were approximately 60% for sexual transmission and 95% for transmission through shared needles during intravenous drug injection [16]. All these results would suggest that, for a part of the respondents at least, there was some confusion between HBV and Hepatitis C Virus (HCV), an infection which is principally transmitted through intravenous drug use and, very rarely, through sexual relations. The probable difficulty people have in differentiating between the various types of hepatitis (described elsewhere [13,14,17]) makes any interpretation of the indicator “knowledge about hepatitis B transmission modes” complex. This is also true for the indicator’s associations with the other variables studied here, including perceptions. The high level of fear reported by the respondents in our study with respect to viral hepatitis was previously observed in a French study carried out in 2006 [14].

In the present study, previous screening for HBV during one’s lifetime was reported 2.2 times less often than HIV screening. This is probably an underestimation as, in France, in 2010, according to the data provided by laboratories [22,23], the number of HBV tests performed was only 1.5 times lower than the number of HIV tests. Moreover, the proportion of women who had a child less than one year old and who declared that they had previously been screened for HBV was low (40%) despite the fact that such screening - obligatory during pregnancy - was performed in 87% of women who gave birth in 2009 (from Certificats de Santé du 8ème jour - health certificates which must be completed before the child is eight days old – data not published). This last observation may however be explained by the possible lack of specific information provided by health professionals to pregnant women when prescribing HBV screening. In multivariate analyses, HBV screening was more often declared by those who perceived themselves to be at higher risk than the average person, by those born in zones with high HBV endemicity, and by those who declared intravenous drug use in their lifetime. The level of screening reported by the second and third of these three populations – both target populations for recommended screening [7,8] – would nevertheless seem to be very insufficient (45%), despite possible under-reporting. This value can be compared with the numbers of French general practitioners in 2009 who reported that they systematically proposed HBV screening to these same two high risk populations (39% and 73%, respectively) [24]. Independently of other factors, in particular educational and income levels, HBV screening was more frequently reported by inhabitants of Île-de-France. This trend was also observed in multivariate analyses for HIV (data not shown) as well as in numbers of HIV and HBV screening tests performed in laboratories [22,23] might be linked to a better access to screening in Île-de-France.

In our study, the proportion of people declaring that they currently had or that they had previously had hepatitis B (3.7%) is coherent with the estimated seroprevalence of anti-HBc antibodies (7.3%) from a survey performed in 2004 on a random sample of the general French metropolitan population if we take into account the proportion of infected people who were aware of their status (45%) [2]. The proportion of people declaring that they currently had or that they had previously had hepatitis B was higher in those who reported intravenous drug use and increased with the level of HBV endemicity in the country of birth, which is also in line with the results from the 2004 prevalence survey [2].

We found that 47% of the respondents reported being vaccinated against hepatitis B. This value is very similar to that found in the Baromètre Santé 2010 survey, which was carried out in France in 2010 using a similar methodology (48%) (data not published). In multivariate analysis, anti-HBV vaccination was most often reported by those people who believed that they were at higher risk than the average person of being infected. Moreover, vaccination was more common in the 18–30 year-old population than in older populations. This result was also observed in a study in Germany [16]. Indeed, during their adolescent years between 1995 and 1998, young adults were the population with the highest proportion of vaccination, before vaccination campaigns were stopped in schools in 1998 [11,25].

The absence of any association between reported vaccination and intermediate or high HBV endemicity in one’s country of birth as well as between reported vaccination and reporting intravenous drug use at least once during one’s lifetime suggests that there is insufficient anti-HBV vaccination coverage in these populations at highest risk [6]. This result is in line with data provided from French mandatory notification of acute hepatitis B which indicated that at least 53% of reported cases between 2003 and 2011 corresponded to a vaccine indication [25]. This low level of reported vaccine coverage can be viewed in the light of the difficulty which French general practitioners have approaching the subject of drug use with patients (41% in 2009) and in proposing screening before vaccination in people born in zones of high HBV endemicity [24,26].

Multivariate analyses highlight an association between educational level and HBV “knowledge of transmission modes” and “screening and vaccination practices” indicators. Accordingly, they underline the necessity to reduce social inequalities in health. This is all the more important since the prevalence of hepatitis B is higher in individuals in an economically and socially vulnerable situation [2].

One must be careful when interpreting the results of this study. As with any self-reported study, certain biases - notably memory bias and social desirability bias - cannot be excluded. Nevertheless these results show the perception and the representation of the respondents. The different types of viral hepatitis are not known very well by quite a large part of the general population [13,14,16,17,21]. Consequently, any interpretation of the specific values of knowledge and perception indicators is complex. What is interesting however is that the values of these indicators can be compared with those for similar indicators associated with other diseases (in the present study we did so with HIV) and can be followed over time. The coherence between the associations highlighted in the present study and those which have been found elsewhere (for example the higher reported prevalence of HBV infection in persons at highest risk) strengthens the validity of our results. Finally, in this study, the participation rate (about 66%) was rather good and close to those observed in other random sampling telephone surveys [27] and data were adjusted for socio-demographic data (age, gender, living in couple and professional activity). However, some people were certainly underrepresented, in particular migrants in the most vulnerable economic and social situations (given that being a French speaker was one of the criteria for eligibility) and consequently it is possible that the reported prevalence of infection was underreported [2].

## Conclusions

This study provided, for the first time in France, quantitative data on the knowledge, perceptions and practices of the general population with respect to hepatitis B. It highlights a lower level of knowledge about the disease together with less screening in the general population when compared to HIV. Furthermore, it suggests that there is confusion in the general population about the differences between hepatitis B and C. More widely, although France is known to be the country with the best hepatitis care delivery in Europe [28], improvement is needed, in particular concerning HBV screening. Indeed, 55% of HBV-infected persons were unaware of their status in general population in 2004, this proportion reaching 80% among individuals born in high HBV endemic countries (Asia, Sub Saharan Africa) among whom HBV prevalence was estimated at 4% [2]. In comparison, about 19% of persons living with HIV were undiagnosed [29]. Moreover, despite recent progress in HBV vaccine coverage in toddlers, situation is still critical for teenagers aged of 15 years (< 50%) who will begin their sexual life [12]. All these results demonstrate that there is room for improvement in HBV, notably in comparison to HIV.

The results of the present study should prove useful for interventions targeted at healthcare professionals and for future information and prevention campaigns directed at the general population and populations at highest risk of HBV [24,26] even if communication is not sufficient in itself [30]. It would certainly seem important to improve public understanding of the specificities of both hepatitis B and C in order to contribute to improvement of screening and vaccination practices.

## Competing interests

The authors declare that they have no competing interests.

## Authors’ contributions

CB and AG conducted the data analysis, the literature review and wrote the manuscript. NB designed and coordinated the study and helped in the writing of the manuscript. LS contributed to data analysis. LS, CJ, CS and NB interpreted the results and commented on various drafts of the article. The “KABP France” group provided useful advice on the paper’s content and commented on final draft of the paper. All authors participated in the design of the KABP 2010 survey. All authors approved the final version and accept responsibility for the paper.

## Pre-publication history

The pre-publication history for this paper can be accessed here:

http://www.biomedcentral.com/1471-2458/13/576/prepub
